# MRI visibility and displacement of elective lymph nodes during radiotherapy in head and neck cancer patients

**DOI:** 10.3389/fradi.2022.1033521

**Published:** 2022-11-03

**Authors:** Floris C. J. Reinders, Peter R. S. Stijnman, Mischa de Ridder, Patricia A. H. Doornaert, Cornelis P. J. Raaijmakers, Marielle E. P. Philippens

**Affiliations:** Department of Radiotherapy, University Medical Centre Utrecht, Utrecht, Netherlands

**Keywords:** magnetic resonance imaging, lymph nodes, radiotherapy, squamous cell carcinoma of head and neck, head and neck neoplasm, elective neck irradiation, elective treatment

## Abstract

**Background and purpose:**

To decrease the impact of radiotherapy to healthy tissues in the head and neck region, we propose to restrict the elective neck irradiation to elective lymph nodes at risk of containing micro metastases instead of the larger lymph node volumes. To assess whether this new concept is achievable in the clinic, we determined the number, volume changes and displacement of elective lymph nodes during the course of radiotherapy.

**Materials and methods:**

MRI scans of 10 head and neck cancer (HNC) patients were acquired before radiotherapy and in week 2, 3, 4 and 5 during radiotherapy. The weekly delineations of elective lymph nodes inside the lymph node levels (Ib/II/III/IVa/V) were rigidly registered and analyzed regarding number and volume. The displacement of elective lymph nodes was determined by center of mass (COM) distances, vector-based analysis and the isotropic contour expansion of the lymph nodes of the pre-treatment scan or the scan of the previous week in order to geographically cover 95% of the lymph nodes in the scans of the other weeks.

**Results:**

On average, 31 elective lymph nodes in levels Ib-V on each side of the neck were determined. This number remained constant throughout radiotherapy in most lymph node levels. The volume of the elective lymph nodes reduced significantly in all weeks, up to 50% in week 5, compared to the pre-treatment scan. The largest median COM displacements were seen in level V, for example 5.2 mm in week 5 compared to the pre-treatment scan. The displacement of elective lymph nodes was mainly in cranial direction. Geographical coverage was obtained when the lymph node volumes were expanded with 7 mm in case the pre-treatment scan was used and 6.5 mm in case the scan of the previous week was used.

**Conclusion:**

Elective lymph nodes of HNC patients remained visible on MRI and decreased in size during radiotherapy. The displacement of elective lymph nodes differ per lymph node level and were mainly directed cranially. Weekly adaptation does not seem to improve coverage of elective lymph nodes. Based on our findings we expect elective lymph node irradiation is achievable in the clinic.

## Introduction

Most head and neck cancer (HNC) patients experience high rates of long-term complications such as xerostomia, dysphagia, carotid stenosis and hypothyroidism (1–4) when treated with radiotherapy. Radiotherapy consists of a high radiation dose directed to the primary tumor and the lymph nodes with metastases, whereas a lower radiation dose is directed to the large lymph drainage volumes on both sides of the neck. The lymph drainage volumes are categorized in different lymph node levels (Ib to V) based on anatomical structures, and cover the volumes where microscopical lymph node spread (i.e., occult metastasis) might occur (5). The treatment of occult metastases is also known as elective neck irradiation (ENI). Lymph node levels might be treated too aggressively with ENI, because in the available literature low regional recurrences rates of ≤5% have been reported (6, 7). De-intensifying ENI might lower the complication rates for HNC patients without affecting the oncological effectiveness.

Some studies already decreased the elective dose from approximately 50 Gy to 36–40 Gy using conventional lymph-node levels as target volumes without increasing the regional recurrence rate (8, 9). However, further studies must be awaited (10). The introduction of MRI in the radiotherapy process enables better visualization of elective lymph nodes inside the lymph node levels. The term elective means there is no suspicion of macroscopic tumor based on histology or radiology. However, due to their location we expect that microscopic tumor load (or occult metastases) might be present in these lymph nodes and not in the whole lymph node levels. Therefore, we advocate to only irradiate the elective lymph nodes instead of the larger lymph node levels. With these new smaller target volumes, we aim to (further) reduce the complication rates for HNC patients, without increasing the regional recurrence rate.

In this new ENI concept called elective lymph node irradiation, the lower radiation dose is given to the elective lymph nodes inside the conventional lymph node levels at risk of containing micro-metastases. The dose to the tumor and pathological lymph nodes will remain the same as in the conventional situation. We will explore the elective lymph node irradiation on the MR linac, which integrates an MRI scanner with a linear accelerator. The MR linac enables visualization of elective lymph nodes during radiotherapy. Consequently, the radiation dose to these small targets can be accurately monitored. In our previous treatment planning study, elective lymph node irradiation on the MR-linac was associated with dose reductions >5 Gy in several organs at risk such as the carotid arteries, thyroid and submandibular gland, compared to conventional ENI (11).

Before we can implement elective lymph node irradiation in the clinic, several issues need to be clarified regarding the visibility and displacement of elective lymph nodes during radiotherapy. Firstly, to target the elective lymph nodes, we need to determine if these can be followed on MRI during the full radiotherapy treatment course. Secondly, we need to determine if adaptive radiotherapy is needed and which radiotherapy margins are required to ensure sufficient dose coverage. For this reason, the volume changes and the displacement of elective lymph nodes during radiotherapy need to be examined. Displacement of lymph nodes might occur due to anatomical changes or set-up errors during radiotherapy.

This study describes the detectability, volume changes and displacement of elective lymph nodes in HNC patients during radiotherapy to assess whether elective lymph node irradiation is achievable in the clinic. Therefore, we performed weekly MRIs in HNC patients during radiotherapy and compared segmented volumes of elective lymph nodes in all acquired scans.

## Materials and methods

### Study design, patients and treatment

In this observational imaging study, we included 10 patients with T2-4N0-2M0 histopathological proven squamous cell carcinoma of the oropharynx, hypopharynx or larynx (Table 1). All patients gave written informed consent for another study in which consent was given to use their data within the same research field (METC UMC Utrecht, trial number: NL57164.041.16). Patients were treated with conventional primary radiotherapy which consisted of 30–35 fractions of 2.0–2.3 Gy to the primary tumor, and 1.26–1.55 Gy to the elective lymph node levels (II-IVa). We included additional lymph node levels Ib, IVb, Vab and VIIab as target volumes if indicated by the international guidelines for ENI (5). In some patients (*n* = 3) radiotherapy was combined with concurrent chemotherapy. We administered the radiotherapy on a 6MV conventional linear accelerator (linac) by Volumetric-Modulated Arc Therapy.

**Table 1:** Tumor and treatment characteristics of all patients included in this imaging study.

Treatment and tumor characteristics	*N* (%)
*Tumor location*	
Oropharynx	7 (70%)
Hypopharynx	2 (20%)
Larynx	1 (10%)
*TNM*	
T1	–
T2	3 (30%)
T3	4 (40%)
T4	3 (30%)
N0	3 (30%)
N1	2 (20%)
N2	5 (50%)
N3	–
M0	10 (100%)
M1	–
*Treatment*	
Radiotherapy only	7 (70%)
Radiotherapy + concurrent chemotherapy	3 (30%)

### MRI and registration techniques

All patients underwent MRI one to three weeks before radiotherapy, and during radiotherapy in the middle of weeks 2, 3, 4 and 5 with a variation of +/− 2 days. During scanning all patients were immobilized using a 5-point thermoplastic mask. Elective lymph nodes and lymph node levels were identified with the in-phase and water-only images of the multiple Dixon T2-weighted turbo spin echo (T2 mDixon TSE) scan (TE: 100* *ms, TR: 3,000* *ms, flip angle: 90°, slice thickness: 3* *mm, in plane reconstructed resolution: 0.86–0.94 mm^2^).

**Figure 1:**
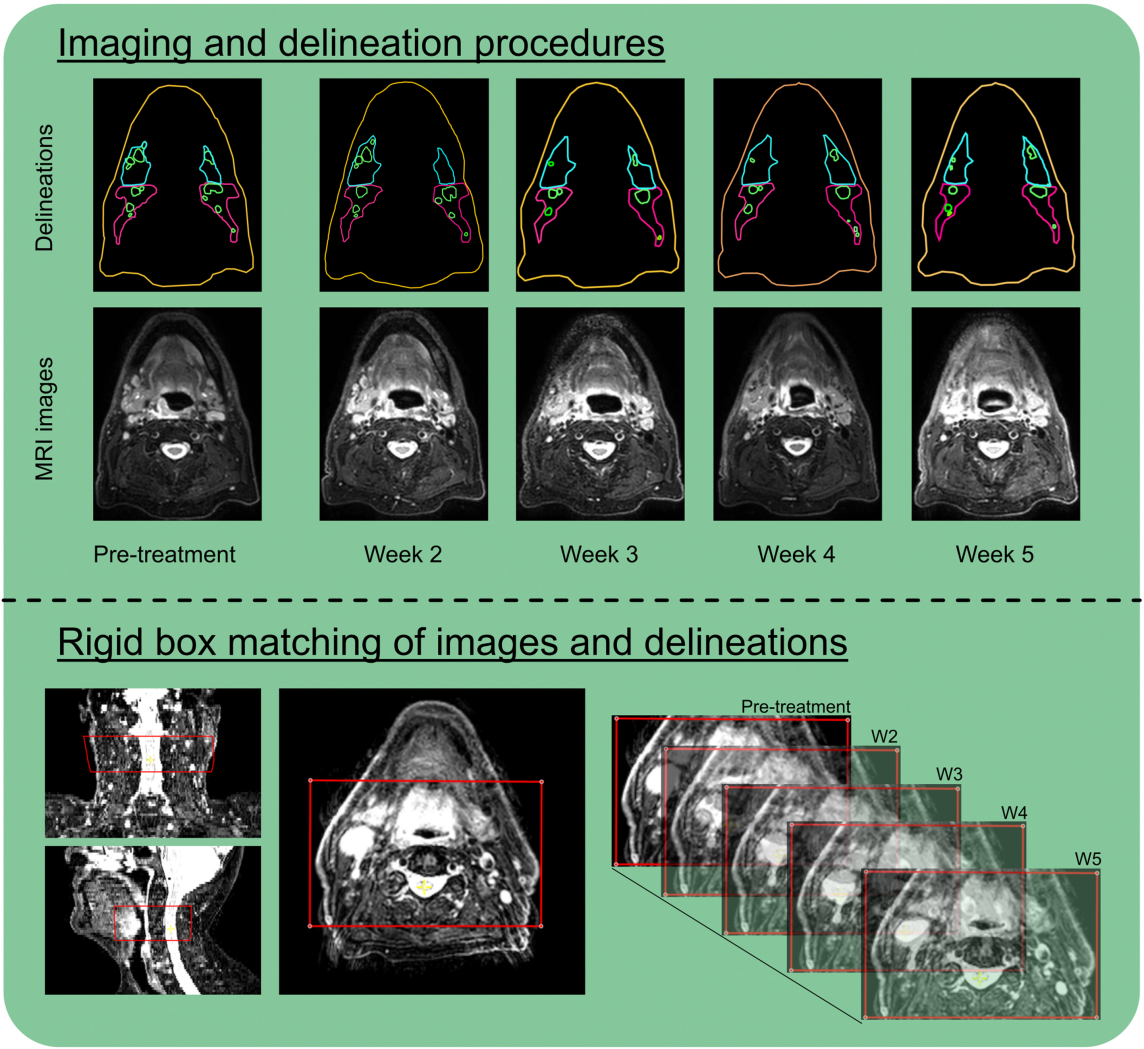
For this study per patient in total 5 MRI scans were obtained in which elective lymph nodes and lymph nodes levels were delineated (top). All scans and delineations were rigidly matched using a box around spine and lymph node levels at the level of the tumor (bottom).

Scans during radiotherapy were rigidly registered to the pre-treatment scan using a box around all lymph node levels and the spine at the level of the primary tumor. With this registration rotational, craniocaudal, ventrodorsal and left-right translations were determined (Figure 1). No deformable matching was used to register MRI scans.

### Definition and delineation of target volumes

Elective lymph nodes were identified and segmented on all MRI scans by one independent observer (medical PhD-student) and checked by a radiation oncologist with over 10 years of experience in the field of HNC. We defined elective lymph nodes as kidney bean shaped, hyperintense structures inside the conventional lymph node levels (Ib-II-III-IVa-V) with a minimal diameter of 2* *mm in the transverse plane. Elective lymph nodes were distinguished from continuous blood vessels with the help of axial and sagittal views. We excluded elective lymph nodes if they were not visible in at least two transverse slices in the pre-treatment scan. Lymph nodes suspect of containing metastases based on imaging or fine needle aspiration were excluded from this analysis.

### Detectability of elective lymph nodes

In every MRI scan, the number of elective lymph nodes was counted and analyzed per lymph node level (Ib to V) on both the ipsilateral and contralateral side. To determine the detectability of elective lymph nodes during the complete course of radiotherapy, we compared the number of elective lymph nodes seen in the pre-treatment scan to the number of elective lymph nodes seen in the scans during radiotherapy.

### Volume of elective lymph nodes

The volumes of elective lymph nodes were compared between the pre-treatment scan and the scans during radiotherapy. Additionally, we analyzed volume differences of elective lymph nodes between the lymph node levels (Ib to V), the side of the neck (ipsilateral vs. contralateral) and irradiation status (irradiated vs. non-irradiated).

### Displacement of elective lymph nodes

The displacement of elective lymph nodes was derived from changes of the center-of-mass (COM) position of elective lymph nodes relative to the pre-treatment scan and to the previous scan. In this way, weekly COM displacements were evaluated on both the ipsilateral and contralateral side. We visualized vector-based 3D-COM displacements in order to detect possible patterns in direction. For the visualization, weekly COM displacements of all lymph nodes were averaged per lymph node level and plotted as a vector with their base set to 0 on the x, y, and z-axis.

### Expansion of elective lymph node contours for geographical coverage

To get an initial impression of a suitable radiotherapy margin for elective lymph node irradiation, lymph node delineations from the pre-treatment scan were isotropically expanded with steps of 1* *mm until 95% of all elective lymph node voxels were geographically covered in the scans during radiotherapy. We defined two expansions to account for set-up errors and anatomical changes during radiotherapy. The first expansion covers 95% of the volume of 95% of all elective lymph nodes in all scans during radiotherapy. This expansion could be used to derive a radiotherapy margin in case the radiotherapy plan will not be adapted during treatment. The second expansion only has to cover 95% of the volume of 95% of all elective lymph nodes of the scan of the previous week. The last margin could be used to derive a margin when the radiotherapy plan on the MR linac will be adapted on a weekly basis.

### Statistical analysis

Ordinal variables were reported as absolute values. Continuous variables were reported as median with inter quartile range (IQR). The lines of the boxes in the boxplots represent the first, second and third quartile. The whiskers represent the 5–95th percentile. Outliers are displayed as dots. We assumed that our data was not normally distributed due to the small sample size. Consequently, we used non-parametric statistical tests. The Friedman test was applied to compare the number of lymph nodes between scans. The volumes of elective lymph nodes between all scans were compared with the Wilcoxon signed rank test. Subgroup analysis regarding the volume differences of lymph nodes was performed with the Mann-Whitney U test in case of a comparison with 2 groups and the Kruskal Wallis test in case of >2 groups. All statistical testing was performed with Statistical Package for Social Sciences (SPSS) version 25 (Released 2017. IBM SPSS Statistics for Windows, Version 25.0. Armonk, NY: IBM Corp.).

## Results

Forty-seven MRI scans of 10 patients obtained before radiotherapy and in weeks 2, 3, 4 and 5 during radiotherapy were included. In three patients a scan was missed in either week 3, 4 or 5. On the available MRI scans, we delineated 595 elective lymph nodes.

### Elective lymph node count

**Figure 2:**
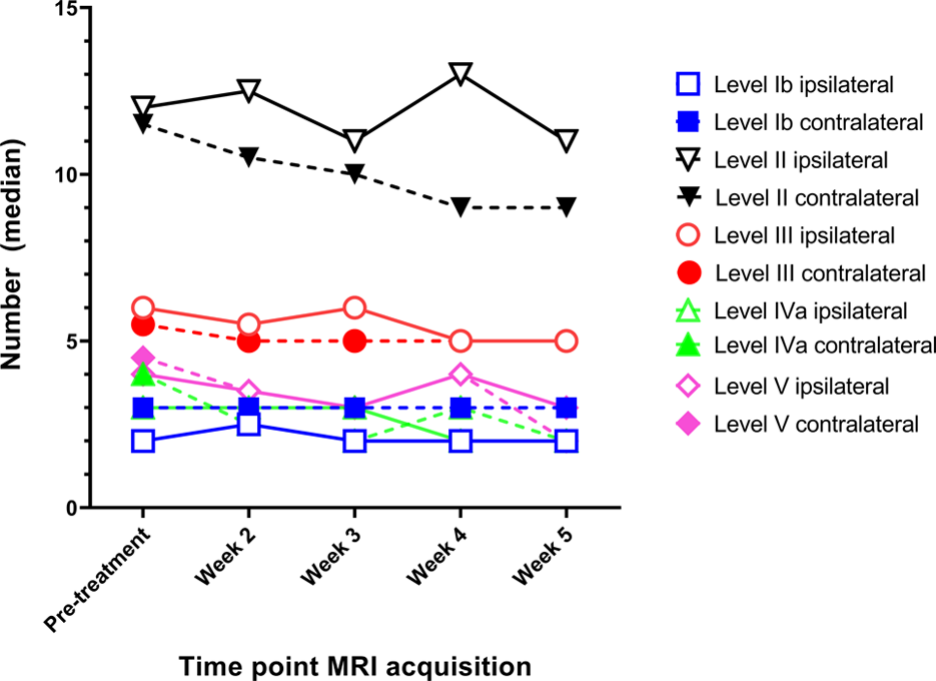
The median number of elective lymph nodes per lymph node level (IB to V) on both the ipsilateral and contralateral side for head and neck cancer patients during the course of radiotherapy.

On average 31 (IQR: 19.8–35.8) elective lymph nodes were visible on MRI per side of the neck. Lymph node level II contained the most lymph nodes on the pre-treatment MRI scan with a median count of 11 (IQR: 8–16) on the ipsilateral side, and 10 (IQR: 5–15) on the contralateral side (Figure 2). Fewer elective lymph nodes were seen on the ipsilateral / contralateral side in lymph node level Ib: 2 / 3 (IQR: 1–3 / 2–4), level III: 6 / 5 (IQR: 4–8 / 4–8), level IV: 3 / 4 (IQR: 3–8 / 3–5) and level V: 4 / 4 (IQR: 2–5 / 2–5).

The lymph node count of elective lymph nodes did not differ throughout the course of radiotherapy treatment, except for the lymph nodes in level II on the contralateral side and level V on the ipsilateral side (Figure 2). The number of lymph nodes reduced significantly with 2.0 lymph nodes in level II (from 11 to 9.0, *p* = 0.029) and 1.0 lymph node in level V (from 4.0 to 3.0, *p* = 0.017). Visual inspection revealed that the lymph node count in level II was mostly decreased by the volume reduction of lymph nodes such that these nodes were not visible anymore in at least two transversal MRI slices. The lymph node count in level V on the other hand was decreased by the displacement of lymph nodes outside the lymph node level.

### Elective lymph node volumes

**Figure 3:**
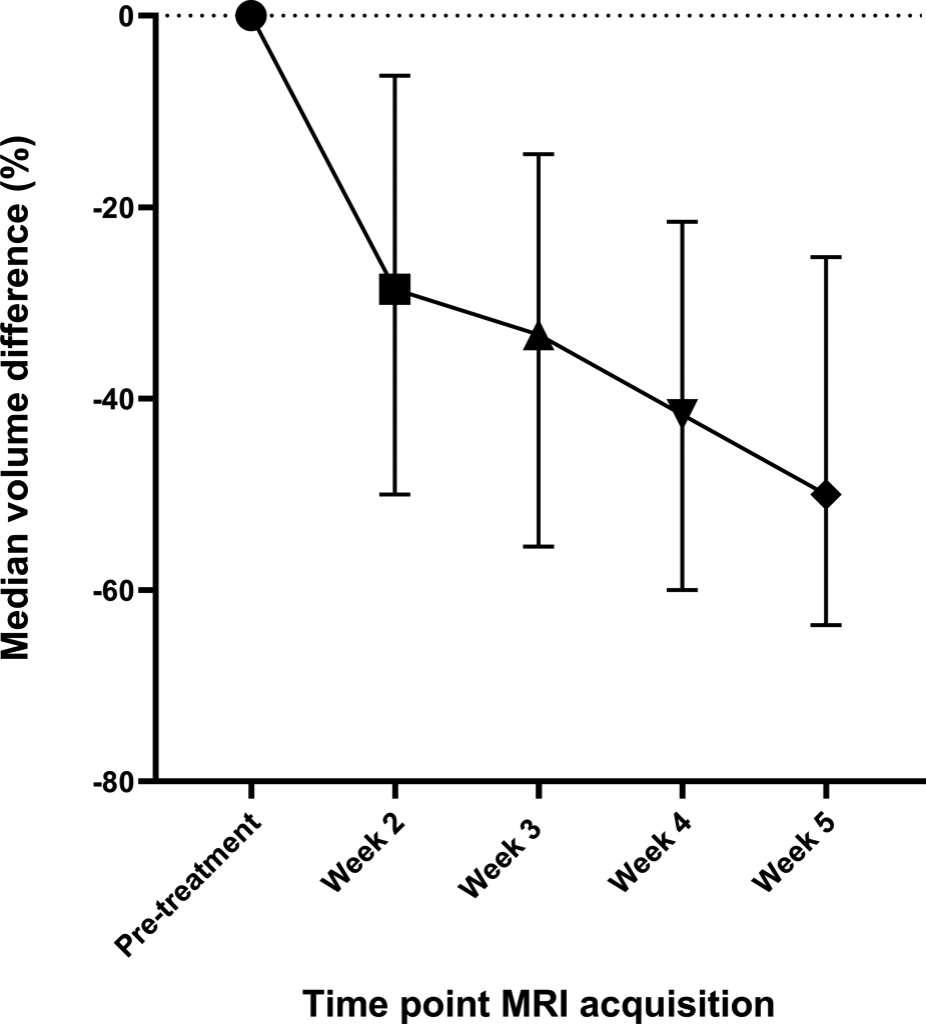
The median differences in the volume of elective lymph nodes during the course of radiotherapy compared to the volume of elective lymph nodes in the pre-treatment scan. The whiskers indicate the interquartile range.

Compared to pre-treatment, the volume of elective lymph nodes reduced significantly in week 2 with −28.6% (IQR: −50.0% to −6.3%, *p *< 0.01), in week 3 with −33.3% (IQR: −55.5% to −14.5%, *p *< 0.01), in week 4 with −41.7% (IQR: −60.0% to −21.5%, *p* < 0.01) and in week 5 with −50.0% (IQR: −63.6% to −25.2%, *p* < 0.01) (Figure 3).

In all weeks, the volume of lymph nodes in lymph node levels I and V decreased more compared to the volume of lymph nodes in other lymph node levels (Supplementary File 1). In week 4 and 5, the volume of elective lymph nodes located at the ipsilateral side decreased more compared to the volume of lymph nodes on the contralateral side (Supplementary File 2). At all time points, the volume of elective lymph nodes that were irradiated was not significantly different from the volume of non-irradiated lymph nodes (Supplementary File 3).

### Displacement of elective lymph nodes

**Figure 4:**
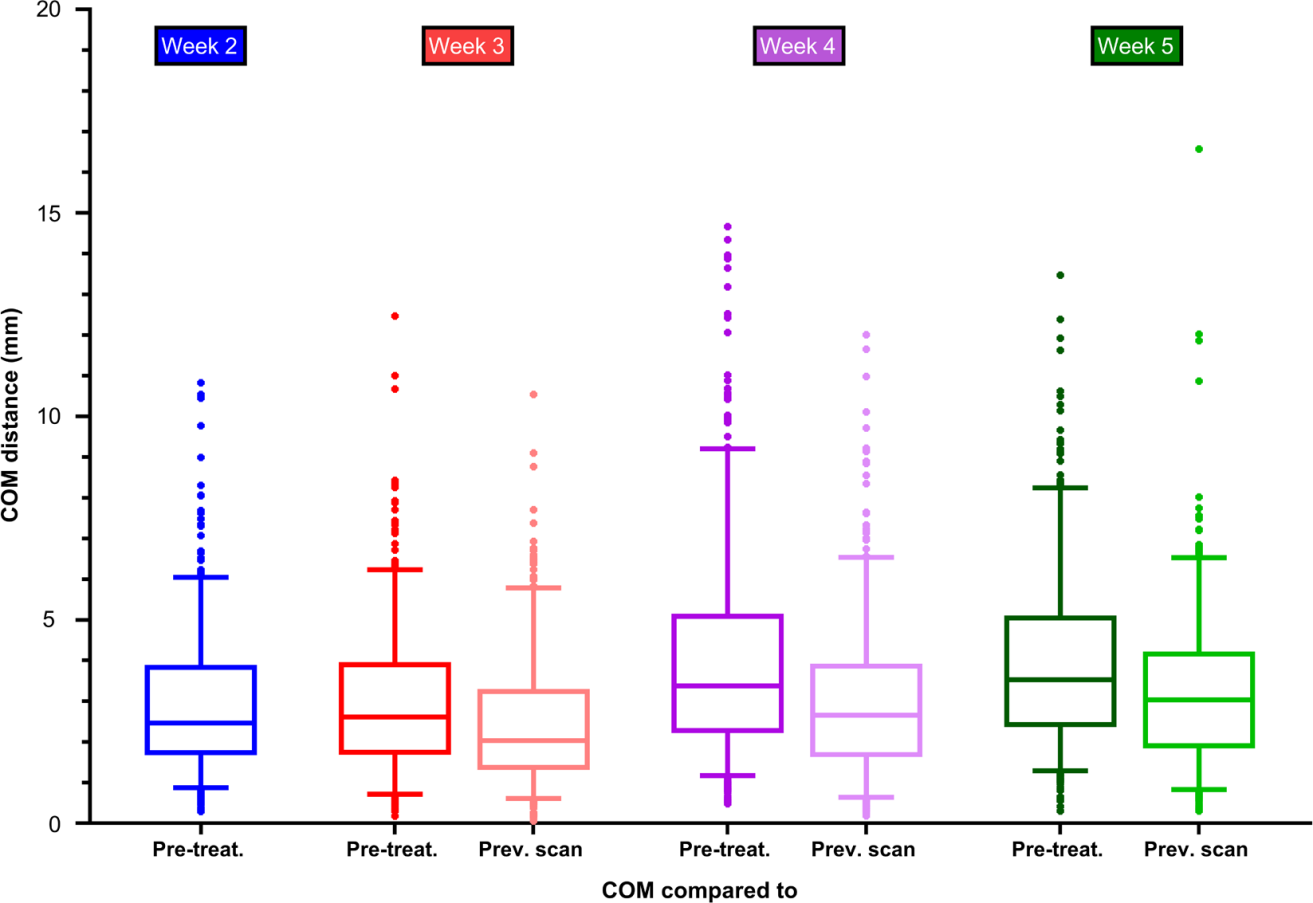
Centre of mass distance between delineations of elective lymph nodes in the pre-treatment scan and the delineations in the scans of week 2, 3, 4 and 5 during radiotherapy. The centre of mass was compared to the pre-treatment scan and to the previous scan.

Median COM distances between lymph nodes in the pre-treatment scan and in the scans at week 2, 3, 4 and 5 were: 2.5 mm (IQR: 1.7–3.9), 2.6 mm (IQR: 1.7–3.9), 3.4 mm (IQR: 2.2–5.1), and 3.5 mm (IQR: 2.4–5.1). The median COM distances reduced when delineations were compared to the previous scan. These values amounted to 2.0 mm (IQR: 1.3–3.3) between week 2 vs. 3, 2.7 mm (IQR: 1.6–3.9) between week 3 vs. 4 and 3.0 mm (IQR: 1.9–4.2) between week 4 vs. 5 (Figure 4). In all weeks, the largest COM distances were observed in lymph nodes located in lymph node level V (Supplementary file 4). The mean displacement vectors of the lymph nodes in levels all indicated a displacement in cranial direction with respect to the pre-treatment position most clearly observed in level III and IV. The displacements in the axial/transverse plane of the lymph nodes in all lymph node levels were more randomly distributed (Supplementary file 5).

### Elective lymph node contour expansion for geographical coverage

**Figure 5:**
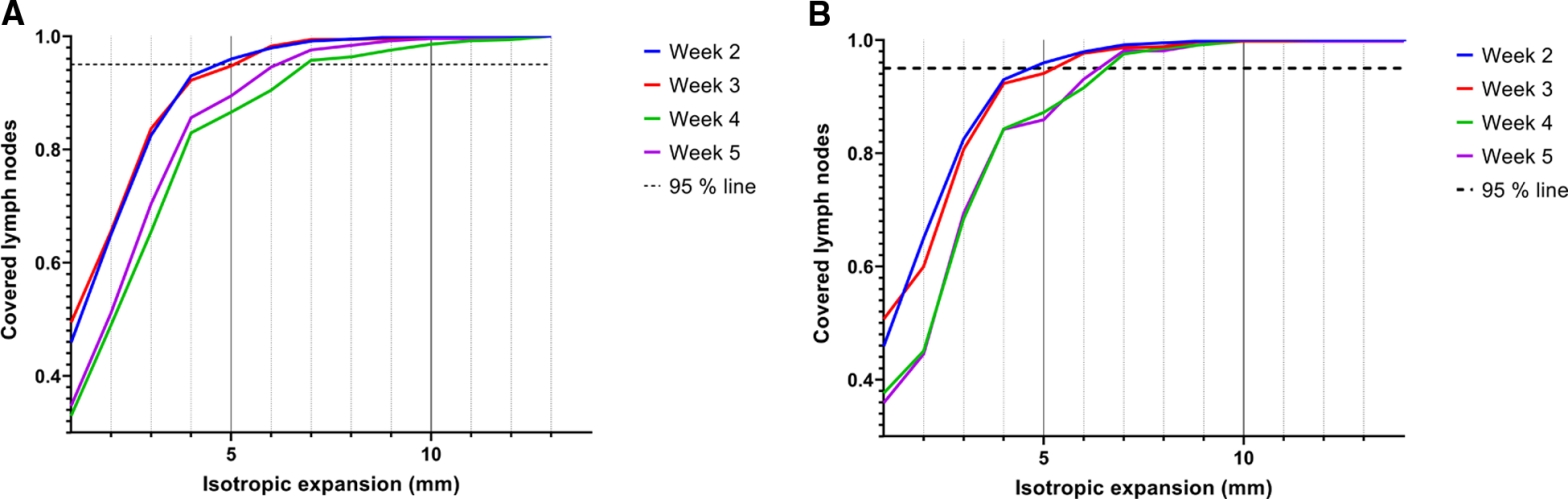
Coverage of elective lymph nodes in the scans of week 2, 3, 4 and 5 when the segmented volumes of the pre-treatment scan (**A**) or the previous scan (**B**) were isotropically expanded. Lymph nodes were considered covered if 95% of all voxels were within the expanded volume.

Geographical coverage was obtained when the lymph node volumes were expanded with an isotropic margin of 5 mm in weeks 2 and 3, and 6–7 mm in weeks 4 and 5 (Figure 5A). The expansion in week 4 decreased with 0.5 mm if the delineations were compared to the delineations of the previous scan (Figure 5B). Especially elective lymph nodes in level V are at risk of not being covered adequately (Supplementary file 6).

## Discussion

In general, elective lymph nodes detectable on MRI before the start of radiotherapy remained visible during radiotherapy. The volume of the elective lymph nodes decreased with approximately 50% during the course of radiotherapy. The displacement of elective lymph nodes was mostly in the cranial direction with median COM displacements of 3.5** **mm in week 5. Geographical coverage was obtained when the elective lymph node delineations were isotropically expanded with 7** **mm in case the pre-treatment scan was used and 6.5** **mm in case the scan of the previous week was used.

Per patient normally 31 elective lymph nodes were detected on each side of the neck. This is comparable to a study that analyzed lymph nodes in healthy volunteers with whole body MRI, with an average of 34 cervical lymph nodes on each side of the neck (12). To this point, no other imaging studies assessed the number, volume or displacement of elective lymph nodes in HNC patients during the course of radiotherapy.

Pathology studies reported a lymph node yield between 34 and 45 in (modified) radical neck dissections on each side of the neck (level Ib-V) (13–16). We reported a lower number of lymph nodes in these levels. However, we have excluded lymph nodes only visible in one transverse MRI slice, lymph nodes with a transverse diameter < 2** **mm and lymph nodes suspect of containing metastases. Also the pathological determination of lymph node yield in neck dissections cannot be considered the gold standard as the lymph node yield is affected by the dissection technique (17) and the person who is performing the dissection (pathologist vs. technician) (15).

In our study the lymph node count remained stable during radiotherapy except for lymph nodes in levels II (−2.0) and V (−1.0). Some lymph nodes in level II were small and only visible in two MRI slices. After the start of radiotherapy these lymph nodes decreased in size and were subsequently only visible in one transversal MRI slice. Unfortunately, lymph nodes in only one transversal slice were not counted by our software we used for this analysis. Lymph nodes in level V were often seen at the border of other lymph node levels (II/III/IV). Therefore, lymph nodes in level V moved from one level to the other. In contrast to our findings, the lymph node yield in (modified) radical neck dissections (levels I-V) was decreased with 7 to 9 lymph nodes if patients were irradiated before surgery (18–20). Radiotherapy is known for its effect on lymph nodes as histological analysis showed significant volume reduction up to −50% after the start of radiotherapy compared to when no radiotherapy is applied (21). The smaller irradiated lymph nodes might be much harder to identify in pathological neck dissections whereas on MRI these lymph nodes might still be visible. This could explain why lymph node yield is further decreased in pathological series than on MRI.

We did not expect the largest volume reductions of lymph nodes to be found in level I and V as these levels are most distant form the primary high dose region. Apparently, the amount of radiation dose does not influence the extent of volume reduction. This is in line with our finding that irradiated lymph nodes do not show larger volume reductions than non-irradiated lymph nodes. An auto-immune response in the whole cervical region induced by the radiotherapy might be the main factor in the reduction of lymph node volumes. Although ipsilateral lymph nodes show significant larger volume reductions than contralateral lymph nodes, these differences were small (largest difference in week 5: −50% ipsilateral vs. −43% contralateral) and might be coincidental.

We found the largest mean COM displacements of elective lymph nodes in level V. Another study assessed mean COM displacements during radiotherapy of separate lymph node levels and reported smaller distances that also were largest in level V: 1.5 ± 0.6** **mm in level Ib, 1.7 ± 0.6** **mm in level II, 2.1 ± 0.9** **mm in level III, 2.9 ± 1.5** **mm in level IV and 5.1 ± 2.0** **mm in level V (22). The smaller COM distances reported in that study might be due to a different rigid registration technique or difference in slice thickness. In our study, MRI scans were rigidly matched using a box containing the lymph node levels and the spine at the level of the primary tumor. A box around the whole elective neck region might have resulted in smaller overall COM displacements of lymph nodes. However, with this alternative matching procedure, the match of the primary tumor might be suboptimal and therefore not suitable for clinical use. Level V is located near the shoulders of the patient where the fixation is less robust than in the more cranial parts. Therefore, larger COM displacements were found in this level. Based on these findings, different radiotherapy margins could be applied for elective lymph nodes based on which lymph node level they are located in.

The COM displacement of elective lymph nodes was mainly in cranial direction compared to the pre-treatment position, where we expected COM displacement towards the center of the body due to fat and muscle loss during radiotherapy. Such specific displacements of elective lymph nodes might have been missed as the rigid registration of MRI scans is not precise enough. Another explanation is that during visual inspection of all scans only one patient showed evident reduction of fat and/or muscle during radiotherapy and therefore we could not demonstrate such an effect. Anisotropic margins might be considered if the overall cranial displacement of lymph nodes can be confirmed in our clinical feasibility study in which the first patients will be treated with elective lymph node irradiation.

To obtain geographical coverage for 95% of all lymph nodes, 7** **mm contour expansion was required in case the pre-treatment scan was used and 6.5** **mm in case the scan of the previous week was used. We were not able to demonstrate a large reduction of the expanded volumes when using weekly plan adaptation. These findings will be evaluated in our clinical feasibility study in which the first patients will be treated with elective lymph node irradiation. During the study daily MRIs will be obtained to see if adaptive strategies are beneficial to reduce margins for elective lymph nodes.

The contour expansions that were determined to obtain geographical coverage are larger than the margins we use in our clinic (3–5** **mm) around the conventional lymph node levels to compensate for random set-up uncertainties (23, 24). Still, the resulting irradiated volume is considerably smaller than with the conventional lymph node level volumes. Dosimetric coverage of the lymph nodes as function of the radiotherapy margin will be further investigated to confirm whether larger margins are necessary for the coverage of elective lymph nodes.

Our study contains several limitations. Firstly, the volume of elective lymph nodes could not be assessed when they were only visible in one transverse slice. Small lymph nodes could therefore be missed in our analysis. Acquiring MRI scans with a thinner slice thickness could have made our observations more precise. However, decreasing the slice thickness with the same signal to noise ratio would have increased the scanning time and consequently also the patient burden. Secondly, we only obtained weekly MRI scans. Intrafraction variations of elective lymph nodes remain unclear and need to be examined before elective lymph node irradiation can be implemented in the clinic. Thirdly, although a large number of lymph nodes was included, only 10 patients were examined in this study.

## Conclusion

In HNC patients during radiotherapy, on average 31 elective lymph nodes on each side of the neck remained visible in most lymph node levels and decreased in size up to −50% in week 5. The displacement of elective lymph nodes was the largest in lymph node level V (5.2** **mm in week 5) and were mainly seen in cranial direction. We were not able to demonstrate a favorable effect of weekly adaptation of the radiotherapy plan for elective lymph node irradiation. Based on our findings we expect elective lymph node irradiation is achievable in the clinic.

## Data Availability

The raw data supporting the conclusions of this article will be made available by the authors, without undue reservation.

## References

[1] Nutting CM Morden JP Harrington KJ Urbano TG Bhide SA Clark C Parotid-sparing intensity modulated versus conventional radiotherapy in head and neck cancer (PARSPORT): a phase 3 multicentre randomised controlled trial. Lancet Oncol. (2011) 12:127–36. 10.1016/S1470-2045(10)70290-4 21236730 PMC3033533

[2] Christianen MEMC Verdonck-De Leeuw IM Doornaert P Chouvalova O Steenbakkers RJHM Koken PW Patterns of long-term swallowing dysfunction after definitive radiotherapy or chemoradiation. Radiother Oncol. (2015) 117:139–44. 10.1016/j.radonc.2015.07.042 26320608

[3] Boomsma MJ Bijl HP Langendijk JA. Radiation-induced hypothyroidism in head and neck cancer patients: a systematic review. Radiother Oncol. (2011) 99:1–5. 10.1016/j.radonc.2011.03.002 21459468

[4] Wilbers J Dorresteijn LD Haast R Hoebers FJ Kaanders JH Boogerd W Progression of carotid intima media thickness after radiotherapy: a long-term prospective cohort study. Radiother Oncol. (2014) 113:359–63. 10.1016/j.radonc.2014.10.012 25466374

[5] Grégoire V Ang K Budach W Grau C Hamoir M Langendijk JA Delineation of the neck node levels for head and neck tumors: a 2013 update. DAHANCA, EORTC, HKNPCSG, NCIC CTG, NCRI, RTOG, TROG consensus guidelines. Radiother Oncol. (2014) 110:172–81. 10.1016/j.radonc.2013.10.010 24183870

[6] Kaanders JHAM van den Bosch S Dijkema T Al-Mamgani A Raaijmakers CPJ Vogel WV. Advances in cancer imaging require renewed radiotherapy dose and target volume concepts. Radiother Oncol. (2020) 148:140–2. 10.1016/j.radonc.2020.04.016 32361663

[7] Van Den Bosch S Dijkema T Verhoef LCG Zwijnenburg EM Janssens GO Kaanders JHAM. Patterns of recurrence in electively irradiated lymph node regions after definitive accelerated intensity modulated radiation therapy for head and neck squamous cell carcinoma. Int J Radiat Oncol Biol Phys. (2016) 94:766–74. 10.1016/j.ijrobp.2015.12.002 26972649

[8] Deschuymer S Nevens D Duprez F Daisne J-F Dok R Laenen A Randomized clinical trial on reduction of radiotherapy dose to the elective neck in head and neck squamous cell carcinoma; update of the long-term tumor outcome. Radiother Oncol. (2020) 143:24–29. 10.1016/j.radonc.2020.01.005 32044165

[9] Maguire PD Neal CR Hardy SM Schreiber AM. Single-arm phase 2 trial of elective nodal dose reduction for patients with Locoregionally advanced squamous cell Carcinoma of the head and neck. Int J Radiat Oncol Biol Phys. (2018) 100:1210–6. 10.1016/j.ijrobp.2017.12.277 29452770 PMC6062207

[10] van den Bosch S Dijkema T Kunze-Busch MC Terhaard CHJ Raaijmakers CPJ Doornaert PAH Uniform FDG-PET guided GRAdient Dose prEscription to reduce late Radiation Toxicity (UPGRADE-RT): study protocol for a randomized clinical trial with dose reduction to the elective neck in head and neck squamous cell carcinoma. BMC Cancer. (2017) 17:1–8. 10.1186/s12885-017-3195-7 28327089 PMC5361684

[11] Reinders FCJ Heijst Tv Mases J Terhaard CHJ Doornaert PAH Philippens MEP Magnetic resonance guided elective neck irradiation targeting individual lymph nodes: a new concept. Phys Imaging Radiat Oncol. (2021) 20:76–81. 10.1016/j.phro.2021.10.006 35169639 PMC8829887

[12] Donners R Yiin RSZ Blackledge M Koh DM. Whole-body diffusion-weighted MRI of normal lymph nodes: prospective apparent diffusion coefficient histogram and nodal distribution analysis in a healthy cohort. Cancer Imaging. (2021) 21:1–10. 10.1186/s40644-021-00432-4 34838136 PMC8627090

[13] Nurimba M Hines W Sinha U Mathew A Kokot N Swanson M. Evaluation of lymph node ratio and lymph node yield as prognosticators of locoregional recurrence in p16-associated oropharyngeal squamous cell carcinoma. Head Neck. (2020) 42:2811–20. 10.1002/hed.26324 32542889 PMC8559522

[14] Sheppard SC Frech L Giger R Nisa L. Lymph node yield and ratio in selective and modified radical neck dissection in head and neck cancer—impact on oncological outcome. Cancers (Basel). (2021) 13(9):2205. 10.3390/cancers13092205 PMC8125696 34064344

[15] Marres CCM De Ridder M Hegger I Van Velthuysen MLF Hauptmann M Navran A The influence of nodal yield in neck dissections on lymph node ratio in head and neck cancer. Oral Oncol. (2014) 50:59–64. 10.1016/j.oraloncology.2013.09.014 24161464

[16] Iocca O Di Maio P De Virgilio A Pellini R Golusiński P Petruzzi G Lymph node yield and lymph node ratio in oral cavity and oropharyngeal carcinoma: preliminary results from a prospective, multicenter, international cohort. Oral Oncol. (2020) 107:104740. 10.1016/j.oraloncology.2020.104740 32380357

[17] Devaraja K Pujary K Ramaswamy B Nayak DR Kumar N Nayak D. Lymph node yield in treatment naïve cases of head and neck squamous cell carcinoma: en bloc lymphadenectomy versus level-by-level dissection. J Laryngol Otol. (2021) 135:359–66. 10.1017/S0022215121000621 33715652

[18] Lippert D Dang P Cannon D Harari PM McCulloch TM Hoffman MR Lymph node yield in therapeutic neck dissection: impact of dissection levels and prior radiotherapy. Ann Otol Rhinol Laryngol. (2017) 126:762–7. 10.1177/0003489417730839 28948832

[19] Yu Y Sultana R Rangabashyam MS Mohan N Hwang JSG Soong YL Impact of radiotherapy on neck dissection nodal count in patients with head and neck cancer. Laryngoscope. (2020) 130:1947–53. 10.1002/lary.28620 32401396

[20] Bhattacharyya N. The effects of more conservative neck dissections and radiotherapy on nodal yields from the neck. Arch Otolaryngol - Head Neck Surg. (1998) 124:412–6. 10.1001/archotol.124.4.412 9559688

[21] Gal R. Histological changes in the cervical lymph nodes after radiotherapy. Oncol Rep. (2001) 8:909–11. 10.3892/or.8.4.909 11410808

[22] Tan W Wang Y Yang M Amos RA Li W Ye J Analysis of geometric variation of neck node levels during image-guided radiotherapy for nasopharyngeal carcinoma: recommended planning margins. Quant Imaging Med Surg. (2018) 8:637–47. 10.21037/qims.2018.08.03 30211031 PMC6127525

[23] Astreinidou E Bel A Raaijmakers CPJ Terhaard CHJ Lagendijk JJW. Adequate margins for random setup uncertainties in head-and-neck IMRT. Int J Radiat Oncol Biol Phys. (2005) 61:938–44. 10.1016/j.ijrobp.2004.11.016 15708278

[24] Houweling AC van der Meer S van der Wal E Terhaard CHJ Raaijmakers CPJ. Improved immobilization using an individual head support in head and neck cancer patients. Radiother Oncol. (2010) 96:100–3. 10.1016/j.radonc.2010.04.014 20430462

