# Supplementary material for: MRI visibility and displacement of elective lymph nodes during radiotherapy in head and neck cancer patients
Source: Front Radiol. 2022 Nov 3;2:1033521. doi: 10.3389/fradi.2022.1033521 (PMC10365081; doi:10.3389/fradi.2022.1033521)

Supplementary file 1: lymph node volume during the course of radiotherapy categorized on the lymph node levels.

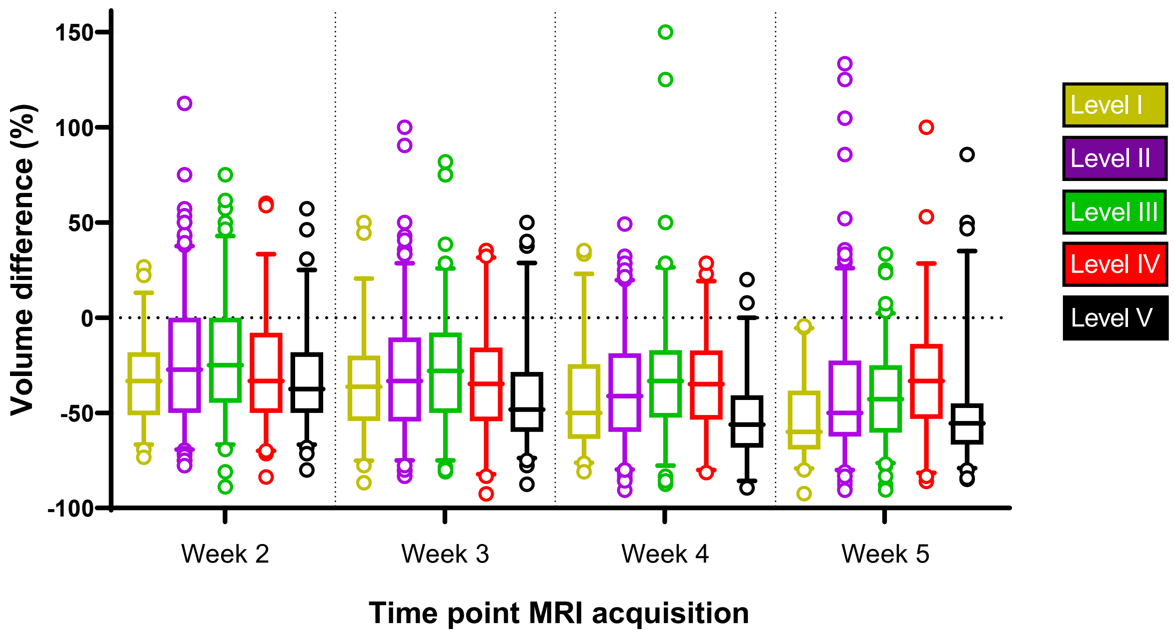

Week 2: volume differences

Lymph node level	I n= 59	II n=254	III n=119	IV n=59	V n=79	Kruskal-Wallis test
Minimum	-73.3	-77.8	-88.9	-83.7	-80.0	p = 0.0182
25% Percentile	-51.3	-50.0	-44.7	-50.0	-50.0	
Median	-33.3	-27.3	-25.0	-33.3	-37.5	
75% Percentile	-18.2	0.0	0.0	-7.9	-18.2	
Maximum	26.7	112.5	75.0	60.0	57.1	
Range	100.0	190.3	163.9	143.7	137.1	
Mean	-33.0	-22.7	-19.5	-28.4	-32.1	
Std. Deviation	24.4	32.1	34.1	31.0	25.6	
Std. Error of Mean	3.2	2.0	3.1	4.0	2.9	

Week 3: volume differences

Lymph node level	I n=54	II n=224	III n=114	IV n=54	V n=66	Kruskal-Wallis test
Minimum	-86.7	-83.3	-81.0	-92.6	-87.5	p = 0.0069
25% Percentile	-54.3	-54.6	-50.0	-54.4	-60.0	
Median	-36.2	-33.3	-27.9	-34.8	-48.3	
75% Percentile	-19.9	-10.4	-7.8	-15.7	-28.6	
Maximum	50.0	100.0	81.8	35.3	50.0	
Range	136.7	183.3	162.8	127.9	137.5	
Mean	-35.0	-28.3	-27.8	-32.8	-42.5	
Std. Deviation	27.6	32.7	32.5	30.8	26.9	
Std. Error of Mean	3.8	2.2	3.0	4.2	3.3	

Week 4: volume differences

Lymph node level	I n=49	II n=223	III n=96	IV n=49	V n=73	Kruskal-Wallis test
Minimum	-81.00	-90.7	-87.5	-81.4	-89.5	p = <0.0001
25% Percentile	-63.7	-60.0	-52.5	-53.6	-68.4	
Median	-50.0	-41.2	-33.3	-34.9	-56.3	
75% Percentile	-24.5	-18.8	-17.1	-17.3	-40.8	
Maximum	35.3	49.1	150.0	28.6	20.0	
Range	116.2	139.8	237.5	110.0	109.5	
Mean	-41.3	-37.6	-30.3	-34.1	-53.3	
Std. Deviation	28.0	28.7	37.4	26.4	22.0	
Std. Error of Mean	4.0	1.9	3.8	3.8	2.6	

Week 5: volume differences

Lymph node level	I n=55	II n=214	III n=105	IV n=50	V n=64	Kruskal-Wallis test
Minimum	-92.4	-90.7	-90.5	-86.1	-85.0	p = 0.0001
25% Percentile	-69.2	-62.5	-60.4	-53.2	-66.7	
Median	-60.0	-50.0	-42.9	-33.3	-55.6	
75% Percentile	-38.3	-22.5	-25.0	-13.8	-45.0	
Maximum	-4.4	133.3	33.3	100.0	85.7	
Range	88.0	224.0	123.8	186.1	170.7	
Mean	-52.2	-39.1	-40.5	-33.1	-50.0	
Std. Deviation	22.6	35.1	25.5	32.8	29.5	
Std. Error of Mean	3.0	2.4	2.5	4.6	3.7	

Supplementary file 2: lymph node volume during the course of radiotherapy categorized on side of the neck (ipsilateral versus contralateral)

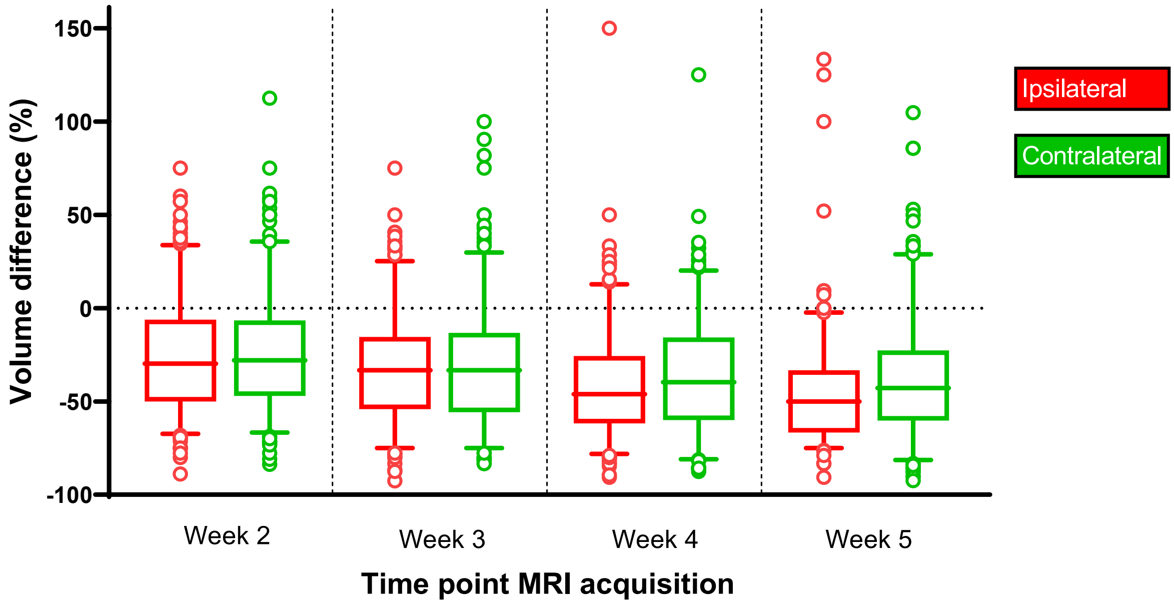

Week 2: volume differences

Side of the neck	Ipsilateral n=290	Contralateral n=280	Mann-Whitney-U test
Minimum	-88.9	-83.7	p = 0.6914
25% Percentile	-50.0	-47.1	
Median	-29.8	-28.0	
75% Percentile	-6.2	-6.5	
Maximum	75.0	112.5	
Range	163.9	196.2	
Mean	-25.7	-24.2	
Std. Deviation	30.7	31.7	
Std. Error of Mean	1.8	1.9	

Week 3: volume differences

Side of the neck	Ipsilateral n=258	Contralateral n=254	Mann-Whitney-U test
Minimum	-92.6	-83.3	p = 0.8460
25% Percentile	-54.2	-55.7	
Median	-33.3	-33.3	
75% Percentile	-15.4	-13.3	
Maximum	75.0	100.0	
Range	167.6	183.3	
Mean	-32.3	-30.1	
Std. Deviation	29.0	33.9	
Std. Error of Mean	1.8	2.1	

Week 4: volume differences

Side of the neck	Ipsilateral n=253	Contralateral n=237	Mann-Whitney-U test
Minimum	-90.7	-87.5	p = 0.0159
25% Percentile	-61.7	-60.0	
Median	-46.2	-39.7	
75% Percentile	-25.7	-15.7	
Maximum	150.0	125.0	
Range	240.7	212.5	
Mean	-41.5	-35.3	
Std. Deviation	29.1	31.1	
Std. Error of Mean	1.8	2.0	

Week 5: volume differences

Side of the neck	Ipsilateral n=250	Contralateral n=238	Mann-Whitney-U test
Minimum	-90.7	-92.4	p = 0.0083
25% Percentile	-66.7	-60.2	
Median	-50.0	-42.7	
75% Percentile	-33.3	-22.6	
Maximum	133.3	104.8	
Range	224.0	197.2	
Mean	-44.9	-38.2	
Std. Deviation	29.6	33.0	
Std. Error of Mean	1.9	2.1	

Supplementary file 3: lymph node volume during the course of radiotherapy categorized on irradiation status (irradiated versus non-irradiated)

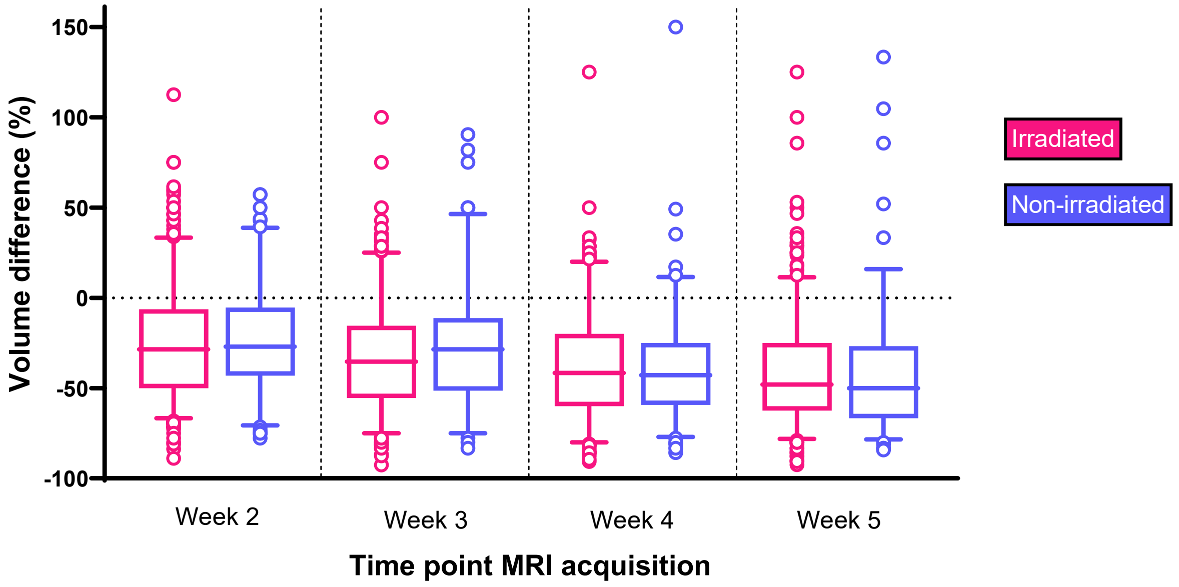

Week 2: volume differences

Irradiation status	Irradiated n=444	Non-irradiated n=126	Mann-Whitney-U test
Minimum	-88.9	-77.8	p = 0.3300
25% Percentile	-50.0	-43.2	
Median	-28.6	-27.1	
75% Percentile	-6.3	-5.4	
Maximum	112.5	57.1	
Range	201.4	134.9	
Mean	-25.5	-23.2	
Std. Deviation	31.3	30.8	
Std. Error of Mean	1.5	2.7	

Week 3: volume differences

Irradiation status	Irradiated n=400	Non-irradiated n=112	Mann-Whitney-U test
Minimum	-92.6	-83.3	p = 0.2080
25% Percentile	-55.6	-51.4	
Median	-35.3	-28.6	
75% Percentile	-15.4	-11.3	
Maximum	100.0	90.5	
Range	192.6	173.8	
Mean	-32.5	-26.5	
Std. Deviation	30.2	35.6	
Std. Error of Mean	1.5	3.4	

Week 4: volume differences

Irradiation status	Irradiated n=379	Non-irradiated n=111	Mann-Whitney-U test
Minimum	-90.7	-85.7	p = 0.9465
25% Percentile	-60.0	-59.3	
Median	-41.7	-42.9	
75% Percentile	-20.0	-25.0	
Maximum	125.0	150.0	
Range	215.7	235.7	
Mean	-38.5	-38.4	
Std. Deviation	29.8	31.7	
Std. Error of Mean	1.5	3.0	

Week 5: volume differences

Irradiation status	Irradiated n=374	Non-irradiated n=114	Mann-Whitney-U test
Minimum	-92.4	-84.2	p = 0.2993
25% Percentile	-62.5	-66.7	
Median	-48.0	-50.0	
75% Percentile	-25.0	-26.7	
Maximum	125.0	133.3	
Range	217.4	217.5	
Mean	-41.5	-42.3	
Std. Deviation	30.1	35.7	
Std. Error of Mean	1.6	3.3	

Supplementary file 4: Centre of mass distance between segmentations of individual lymph nodes in the pre-treatment scan and in the scans of week 2, 3 ,4 and 5 during radiotherapy. The centre of mass was compared to the pre-treatment scan and to the previous scan. The data is categorized based on the lymph node levels.

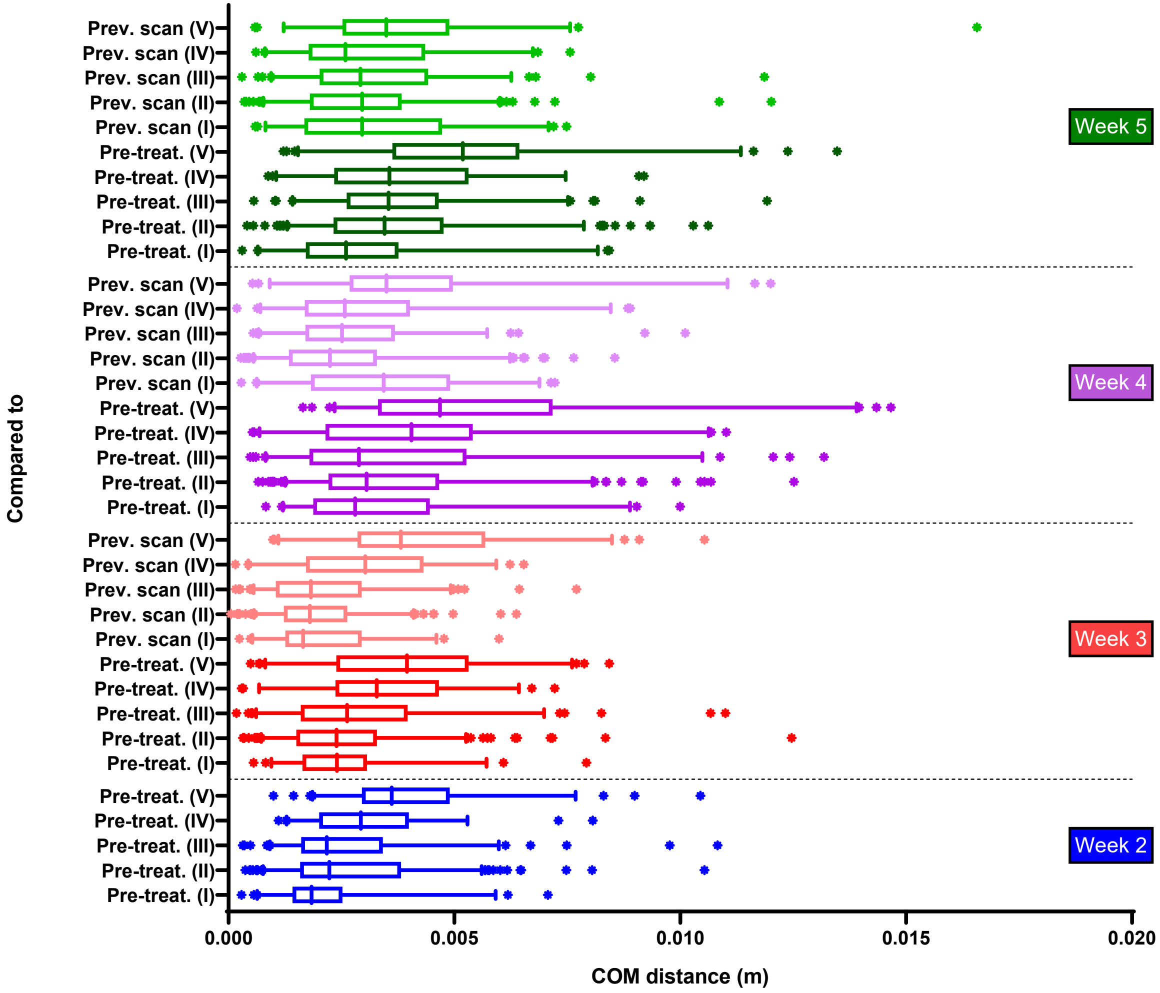

Supplementary file 5: Distribution of the mean of the center of mass distance between segmentations of individual lymph nodes in the pre-treatment scan and in the scans of week 2, 3, 4 and 5 during radiotherapy.

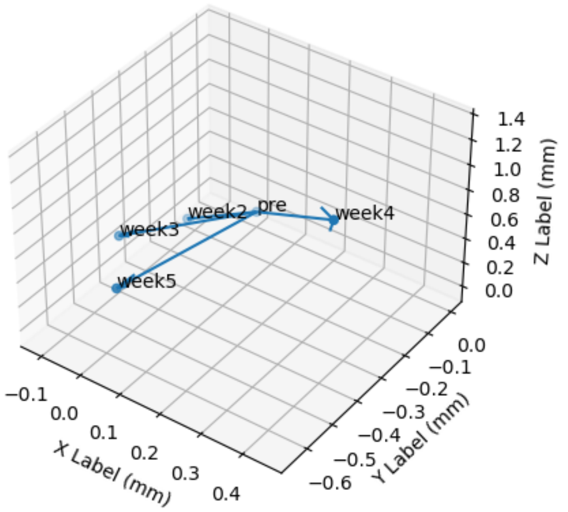

Lymph node level I, right side

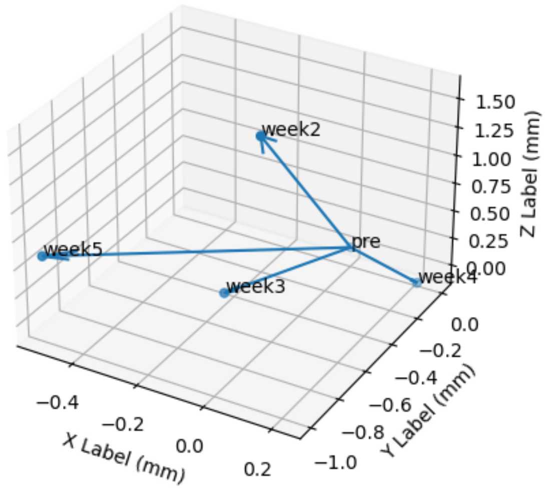

Lymph node level I, left side

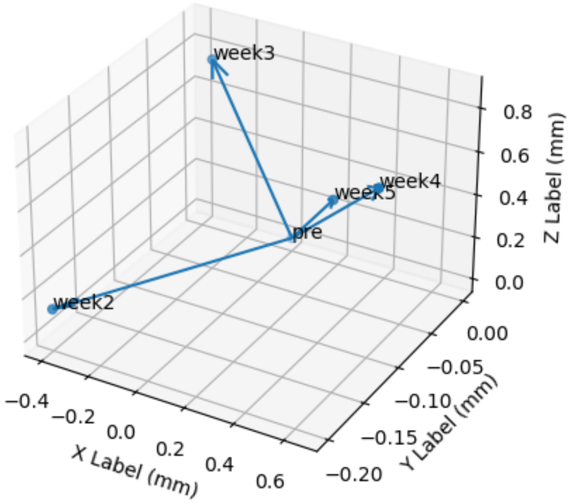

Lymph node level II, right side

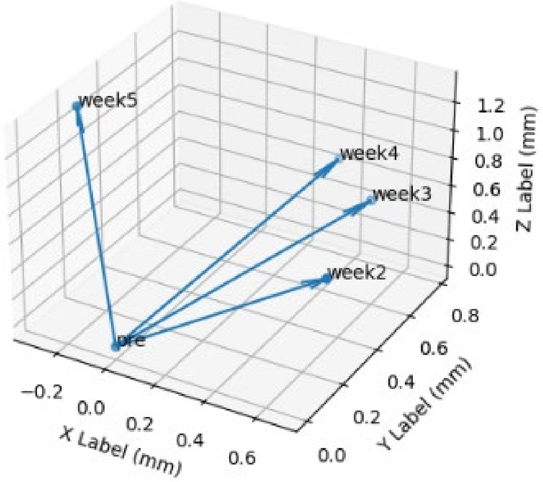

Lymph node level II, left side

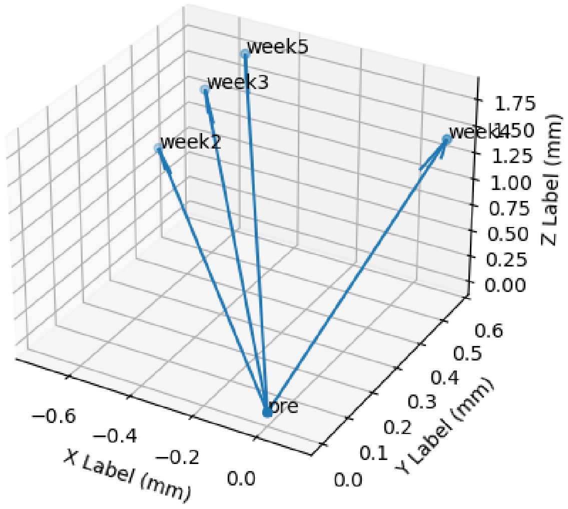

Lymph node level III, right side

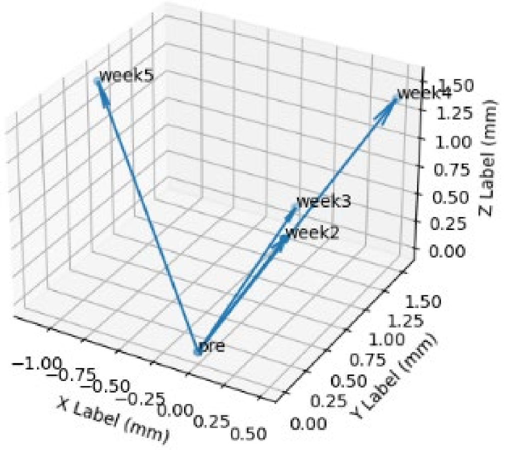

Lymph node level III, left side

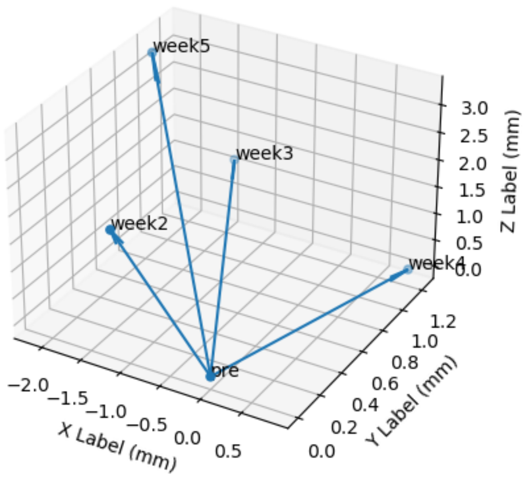

Lymph node level IV, right side

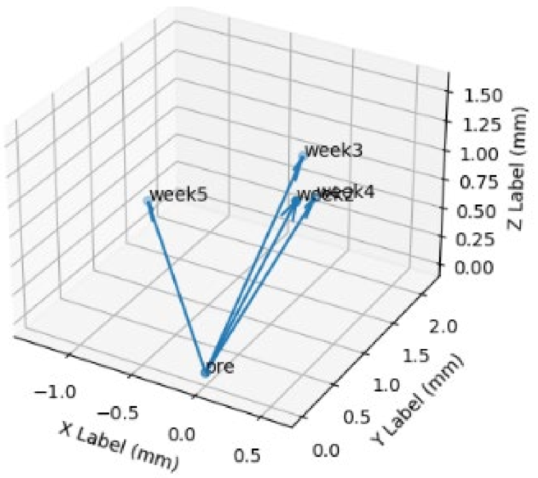

Lymph node level IV, left side

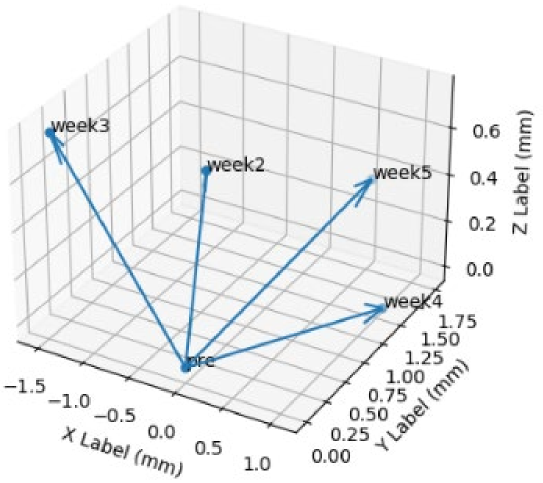

Lymph node level V, right side

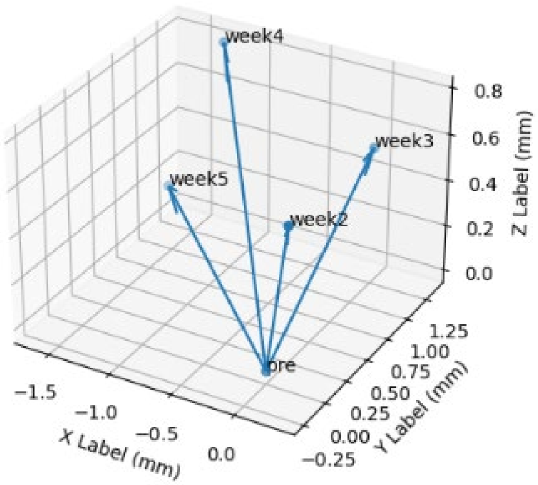

Lymph node level V, left side

Supplementary file 6: Coverage of segmented lymph nodes in the scans of all weeks when the delineations of the pre-treatment scan (A) or the previous scan (B) were isotropically expanded. Lymph nodes were categorized based on the lymph node levels. Lymph nodes were considered covered if 95% of all voxels were within the expanded volume.

(A)

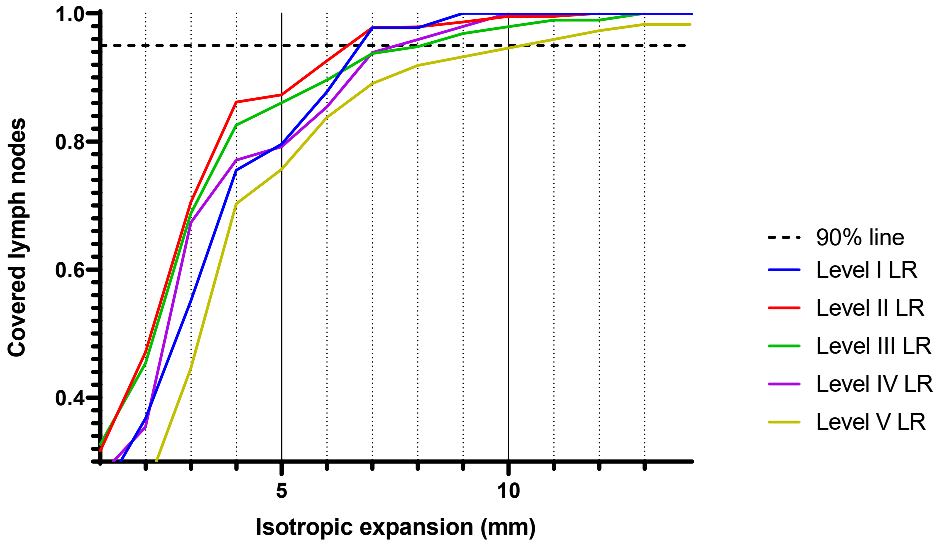

(B)

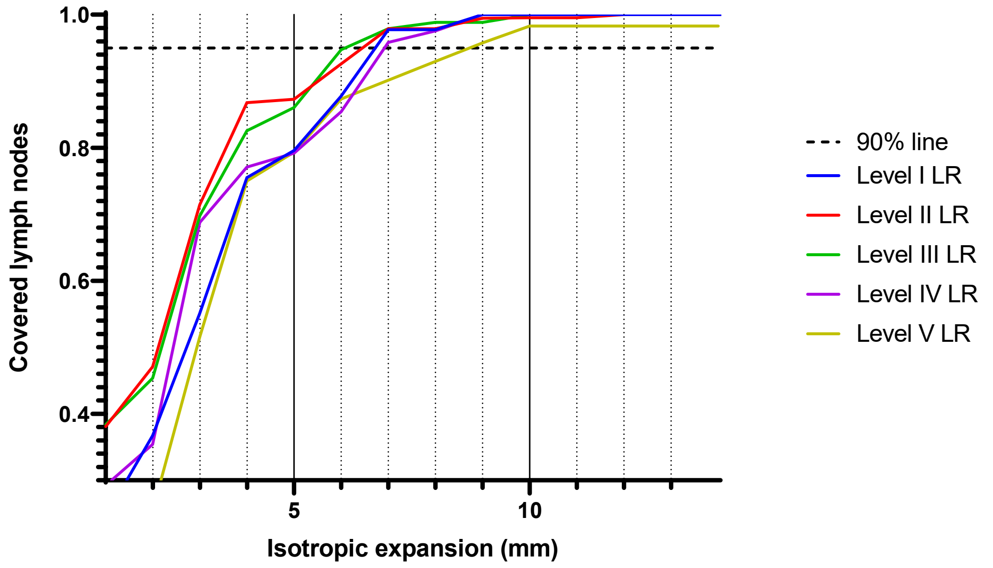

Supplement: Supplementary file 1 [file Datasheet1.pdf]
